# La maladie de pompe de l'adulte: une entité rare à ne pas méconnaitre

**DOI:** 10.11604/pamj.2014.19.304.4501

**Published:** 2014-11-20

**Authors:** Wafa Chebbi, Baha Zantour

**Affiliations:** 1 Service de Médecine Interne, CHU Taher Sfar Mahdia, 5100 Mahdia, Tunisie

**Keywords:** Maladie de pompe, glycogénose, myopathie, histologie, Pompe disease, glycogenolysis, myopathy, histology

## Image en medicine

La myopathie par déficit en maltase acide ou glycogénose de type II décrite d'abord par Pompe en 1932, est la première maladie de surcharge lysosomale identifiée. Il s'agit d'une affection autosomique récessive rare qui atteint classiquement le nouveau-né avec une évolution rapidement mortelle. Les formes tardives débutantes à l’âge adulte sont rares. Nous rapportons l'observation d'un patient âgé de 56 ans qui consultait pour des troubles de la marche d'apparition progressive. L'examen neurologique objectivait une démarche dandinante, en rapport avec un déficit musculaire proximal des membres inférieurs. Le reste de l'examen était sans particularités. Le bilan biologique montrait une élévation modérée du taux de CPK (476 UI/l, normal < 204). Le bilan inflammatoire, l’électrophorèse des protéines sériques, le bilan thyroïdien et les anticorps antinucléaires étaient normaux. L’électromyogramme montrait un tracé de type myogène dans les muscles proximaux. La biopsie musculaire montrait en microscopie optique un aspect typique de myopathie par surcharge glycogénique avec inégalité de la taille des fibres musculaires et présence de vacuoles de tailles variables et positives à la coloration PAS. L’étude enzymatique musculaire confirmait le déficit en maltase acide. L'exploration fonctionnelle respiratoire, le gaz du sang et le scanner thoracique étaient normaux. L’évolution actuelle est caractérisée par une stabilité des symptômes. Le diagnostic de maladie de Pompe de l'adulte doit être évoqué devant une myopathie progressive des ceintures isolée. L'atteinte respiratoire doit être recherchée et surveillée. La biopsie musculaire et l'analyse enzymatique permettent d'en confirmer le diagnostic.

**Figure 1:**
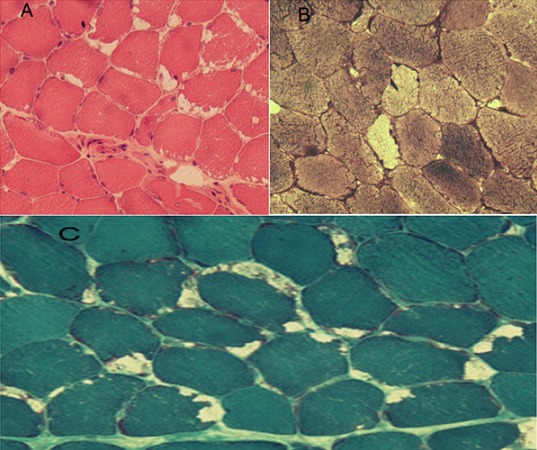
Tissu musculaire prélevé par biopsie A) coloration hématéine-éosine: inégalité de la taille des fibres avec présence de multiples vacuoles de tailles variables; B) coloration PAS: présence de vacuoles de tailles variables PAS+ touchant la majorité des fibres; C) coloration trichrome de Gomori: inégalité de la taille des fibres avec présence de vacuoles de tailles variables

